# Stability and profiling of urinary microRNAs in healthy cats and cats with pyelonephritis or other urological conditions

**DOI:** 10.1111/jvim.15628

**Published:** 2019-11-13

**Authors:** Lisbeth R. Jessen, Lise N. Nielsen, Ida N. Kieler, Rebecca Langhorn, Bert J. Reezigt, Susanna Cirera

**Affiliations:** ^1^ Department of Veterinary Clinical Sciences University of Copenhagen Denmark; ^2^ Blue Star Animal Hospital Gothenburg Sweden; ^3^ Department of Veterinary and Animal Sciences University of Copenhagen Denmark

**Keywords:** Biomarker, CKD, feline, pathogen‐directed microRNA expression, subclinical bacteriuria, ureteral obstruction, UTI

## Abstract

**Background:**

Specific biomarkers of pyelonephritis (PN) in cats are lacking. MicroRNAs (miRNAs) have diagnostic potential in human nephropathies.

**Objectives:**

To investigate the presence/stability of miRNAs in whole urine of cats and the discriminatory potential of selected urinary miRNAs for PN in cats.

**Animals:**

Twelve healthy cats, 5 cats with PN, and 13 cats with chronic kidney disease (n = 5), subclinical bacteriuria (n = 3), and ureteral obstructions (n = 5) recruited from 2 companion animal hospitals.

**Methods:**

Prospective case‐control study. Expression profiles of 24 miRNAs were performed by quantitative PCR (qPCR). Effect of storage temperature (4°C [24 hours], −20°C, and −80°C) was determined for a subset of miRNAs in healthy cats.

**Results:**

Urinary miR‐4286, miR‐30c, miR‐204, miR4454, miR‐21, miR‐16, miR‐191, and miR‐30a were detected. For the majority of miRNAs tested, storage at 4°C and −20°C resulted in significantly lower miRNA yield compared to storage at −80°C (mean log2fold changes across miRNAs from −0.5  ± 0.4 SD to −1.20 ± 0.4 SD (4°C versus −80°C) and from −0.7 ± 0.2 SD to −1.20 ± 0.3 SD (−20°C versus −80°C)). Cats with PN had significantly upregulated miR‐16 with a mean log2fold change of 1.0 ± 0.4 SD, compared with controls (−0.1 ± 0.2, *P* = .01) and other urological conditions (0.6 ± 0.3, *P* = .04).

**Conclusions:**

Upregulation of miR16 might be PN‐specific, pathogen‐specific (*Escherichia coli*), or both.

**Abbreviations**
BSAH: Blue Star Animal Hospital in Gothenburg
CKD: chronic kidney disease
C&S: culture and sensitivity
E. coli: Escherichia coli
ICC: intraclass correlation coefficient
IRIS: International Renal Interest Society
LMM: linear mixed‐effects models
miRNA: microRNA
PN: pyelonephritis
qPCR: quantitative PCR
SB: subclinical bacteriuria
UBC: urine bacterial culture
UCPH: University of Copenhagen
UHCA: University Hospital for Companion Animals, University of Copenhagen
UO: ureteral obstruction

## 1 INTRODUCTION

The diagnosis of bacterial pyelonephritis (PN) in cats can be challenging, and definitive diagnosis requires a positive urine culture obtained by pyelocentesis, an invasive and technically demanding procedure. In practice, diagnosis is often based on compatible clinical, laboratory, and ultrasonographic features along with a positive vesical urine culture. However, the clinical presentation of PN can be nonspecific, and substantial overlap exists in clinical and diagnostic findings between cats with PN and cats with other urological conditions in particular chronic kidney disease (CKD) and ureteral obstruction (UO).^1^

Multiple disease processes can occur concomitantly in the animal,^2^ adding to the challenge of correctly identifying PN without performing pyelocentesis. Furthermore, discrimination between disease conditions is hampered by the common occurrence of subclinical bacteriuria (SB) in cats with CKD^3^ and the phenomenon of cystocentesis culture‐negative pyelonephritis in cats with UO.^4^

MicroRNAs (miRNAs) are small, noncoding RNAs regulating gene expression at the post‐transcriptional level.^5^ In people, tissue miRNAs are investigated as biomarkers of kidney injury^6^, ^7^ and have discriminatory potential in the diagnosis of acute allograft pyelonephritis.^8^ Recent research on miRNA profiling in renal disease has focused on urine as a convenient minimally invasive surrogate for kidney tissue, and differential expression of urinary miRNAs has been identified in cats and dogs with CKD,^9^, ^10^ as well as in people with various renal pathologies, such as acute kidney injury, diabetic nephropathy, IgA nephropathy and lupus nephritis.^7^, ^11^ This indicates that urinary miRNA expression can potentially be used diagnostically as marker of specific diseases, disease severity, or both.

The objectives of the present study were 2‐fold: (1) to investigate the presence and stability of miRNAs in whole urine of cats, and (2) to investigate if selected miRNAs are differentially expressed in cats with PN compared to healthy cats and cats with CKD, UO, SB.

## 2 MATERIALS AND METHODS

### 2.1 Study design

**Figure 1:**
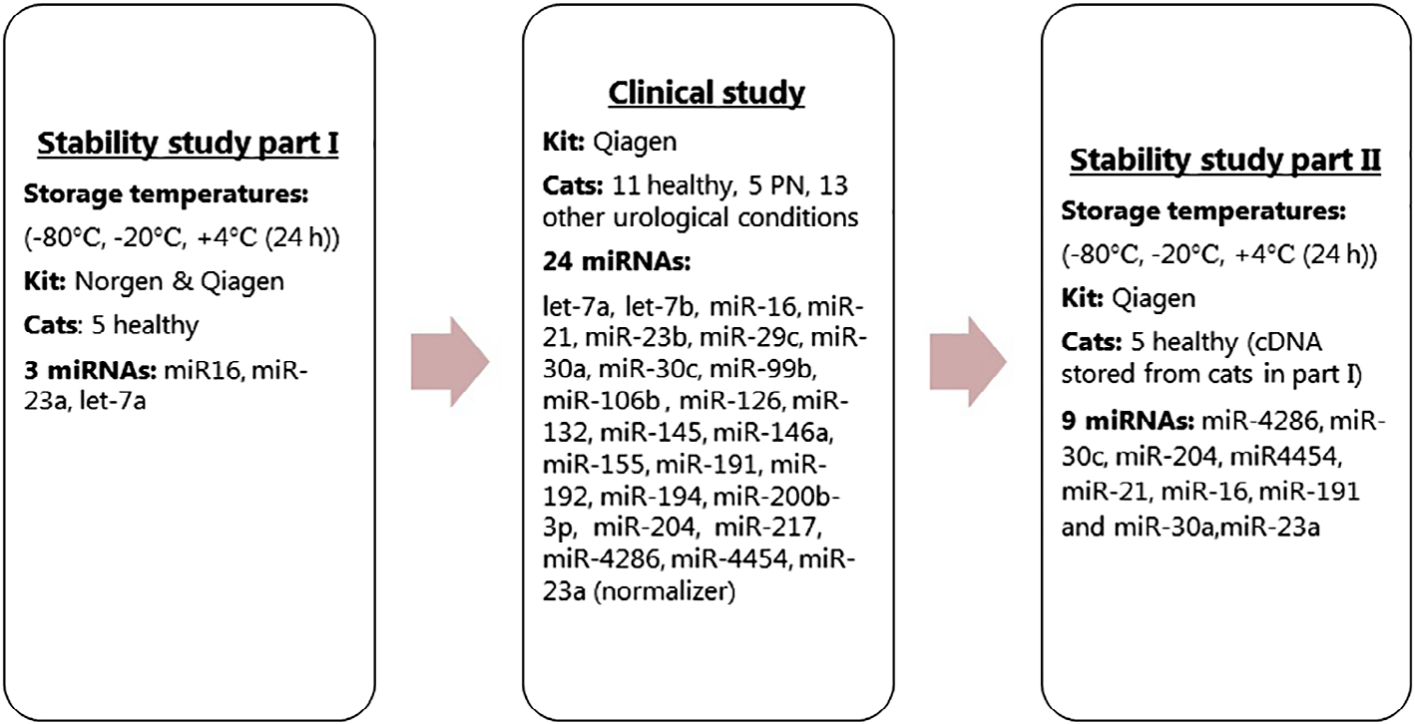
Flow diagram of the study set‐up and miRNAs tested in the different parts of the study

The study was designed as a bicenter prospective case‐control pilot study and divided into 2 substudies: (1) Stability and (2) Clinical study. The stability study investigated the presence and stability of miRNAs in urine of healthy cats and was further subdivided in part I and II. Part I investigated influence of storage temperature on 3 miRNAs, chosen due to high expression in urine, and efficiency of purification of miRNA using 2 different kits Norgen (MicroRNA purification kit, Norgen Biotek Corp, Thorold, ON, Canada) and Qiagen (miRNEasy serum/plasma kit, Qiagen, Hilden, Germany). The clinical study investigated expression profiles of selected miRNAs in healthy cats, cats with PN, and cats with urological conditions other than PN. Part II of the stability study was performed after obtaining the results from the clinical study in order to investigate the influence of storage temperature on the 8 miRNAs expressed in the clinical study (Figure 1).

### 2.2 Recruitment

Cats were recruited from the University Hospital for Companion Animals in Copenhagen (UHCA) and the Blue Star Animal Hospital in Gothenburg (BSAH). Healthy cats, cats with CKD, and cats with SB were recruited prospectively for participation into other ongoing studies in the period 2015 to 2017, and excess urine was used for the present study. Cats with PN and cats with UO were included prospectively into the present study from 2015 to 2017.

Each of the studies was approved by the departmental ethical committee at the University of Copenhagen (UCPH) and informed owner consent for participation was obtained.

### 2.3 Disease categories

Cats were classified into 3 categories: (1) healthy controls, (2) cats with PN, and (3) cats with urological conditions other than PN. Cats with unremarkable clinical and clinicopathological findings were classified as healthy controls. Cats with a positive urine culture obtained by pyelocentesis (pelvic urine culture) or cats with a presentation highly suggestive of PN were classified as PN regardless of whether urological comorbidities were present or not. A clinical presentation highly suggestive of PN was defined as presence of pyrexia, renal pain, azotemia, elevated acute phase proteins combined with a positive cystocentesis urine culture, and resolution of clinical signs and laboratory abnormalities in response to antimicrobial treatment. Cats were classified as having a urological condition other than PN if diagnosed with sterile UO (uni‐ or bilateral ureteral obstructive disease and negative pelvic urine culture), CKD (cats with stable azotemic renal disease [IRIS stage ≥2] and negative cystocentesis urine culture), SB (healthy cats with a positive cystocentesis urine culture in the absence of clinical signs of urinary tract disease), or a combination of the above.

### 2.4 Diagnostic workup

All cats had basic work up performed consisting of clinical examination, hematology, biochemistry, urinalysis, and urine bacterial culture and susceptibility (C&S).

In addition, cats with PN and cats with UO all had abdominal ultrasonography performed, and pyelocentesis was performed in those cats with abnormal dilation (>2 mm) of the renal pelvis.

### 2.5 Urine sampling methods for diagnostic testing and miRNA analyses

Urine for urinalysis and for miRNA analyses was obtained by cystocentesis. Urine for C&S was obtained either by cystocentesis (healthy cats, cats with CKD, SB, and PN) or by pyelocentesis (cats with PN and UO). The majority of cats with UO and PN had dual cystocentesis and pyelocentesis C&S performed.

### 2.6 Microbiology

Quantitative bacterial urine culture and susceptibility testing was performed at the diagnostic laboratories, Sund Vet Diagnostik, Department of Veterinary Disease Biology, UCPH, Denmark and the BSAH diagnostic laboratory, Sweden, respectively. Colony types were identified to the species level by matrix‐assisted laser desorption/ionization time of flight (MALDI‐TOF) mass spectrometry (Vitek MS RUO, BioMérieux, Marcy‐l'Étoile, France). Antimicrobial susceptibility was tested for all isolates using the broth microdilution method (Sensititre Thermo Scientific Fisher, Waltham, Massachusetts) according to the Clinical and Laboratory Standards Institute.^12^, ^13^

### 2.7 Storage conditions

For the stability study, aliquots of whole urine were (1) immediately frozen at −80°C, (2) immediately frozen at −20°C, or (3) refrigerated at +4°C for 24 hours and subsequently frozen at −80°C. Storage conditions for urine samples included in the clinical study are listed in Table 1. Clinical samples from BSAH were transported to the laboratory at UCPH on dry ice (−80°C), and stored for a maximum of 2 years.

**Table 1:** Storage conditions, diagnosis, and characteristics of healthy and diseased cats included in the study

	Healthy controls (N = 12)	PN (N = 5)^a^	Other urological conditions (N = 13)		
			CKD (N = 5)	UO (N = 5)^b^	SB (N = 3)
Age (years)	2 to 16	4 to 11	8 to 17	3 to 10	7 to 10
Breed	8 DSH, 2 Ragdoll, 1 Somali, 1 Norwegian Forest Cat	2 DSH, Maine Coon, 1 Abyssinian, 1 Oriental	4 DSH, 1 Unknown	2 DSH, 1Mix breed, 1 Abyssinian, 1 Oriental	1 DSH, 1 Maine Coon, 1 unknown
Sex	6 FN, 4 MN, 2 F	3 F, 2 FN	3 MN, 1 F, 1 FN	4 MN, 1 F	2 FN, 1 F
Uropathogens	Culture‐negative (cystocentesis)	Pyelocentesis: 3 *Escherichia coli* 1 *Staphylococcus pseudointermedius* Cystocentesis: 1 *Escherichia coli*	Culture‐negative (cystocentesis)	Culture‐negative (pyelocentesis)	1 *Escherichia coli* 1 *Micrococcus Luteus* 1 *Streptococcus spp* (cystocentesis)
Concomitant conditions	—	1 CKD IRIS stage 1 (renal cyst) 1 nonobstructive nephrolithiasis 1 bilateral UO and evidence of CKD (unstaged)	3 IRIS stage II 1 IRIS stage III 1 IRIS stage IV	2 evidence of CKD (un staged)	—
Storage condition	−80	3 samples: −20 1 sample: +4 (24 h) 1 sample: −80	−80	3 samples: +4 (24 h) 2 samples: −80	−80
Recruitment site	UHCA	BSAH 3 UHCA 2	UHCA	UHCA	UHCA

Abbreviations: B&C, bacterial culture and susceptibility testing; BSAH, Blue Star Animal Hospital; CKD, chronic kidney disease; DSH, domestic shorthair; F, female; FN, female neutered; IRIS, International Renal Interest Society; MN, male neutered; PN, pyelonephritis; SB, subclinical bacteriuria; UHCA, University Hospital for Companion Animals; UO, ureteral obstruction.

a PN cats diagnosed by positive culture pyelocentesis for 4 cats and by clinical presentation and positive culture cystocentesis for 1 cat. Two cats with PN had dual cystocentesis and pyelocentesis B&C performed, both with agreeing results.

b Four out of 5 cats with UO had dual cystocentesis and pyelocentesis B&C performed, all with agreeing results.

### 2.8 RNA isolation, cDNA synthesis, and q‐PCR

Two kits for RNA isolation were applied in the stability study part I: (1) The Norgen kit, for which 500 to 800 μL whole urine were used for RNA extraction; and (2) The Qiagen kit, for which 200 μL whole urine were used for RNA extraction. For part II of the stability study and for the clinical study only the Qiagen kit was used. The volume of stored urine from 2 out of 5 cats was inadequate for extraction by both kits at all storage temperatures. Therefore, storage temperatures in part I and part II were compared on duplicates from the following Qiagen samples: 5 samples stored at −20°C, 4 samples stored at −80°C, and 3 samples stored at 4°C. For all samples, 2 independent cDNA syntheses were performed. For part I of the stability study, 2 μL total RNA from Qiagen samples and 4.3 μL total RNA from Norgen samples were used. For the clinical study 10 ng of total RNA isolated with the Qiagen kit were used. The cDNA synthesis was performed according to the method described by Balcells et al^14^ in 2011 with inclusion of a spiked‐in synthetic miRNA (*C. elegans* miR‐39‐3p). Samples were diluted 8‐fold prior to quantitative real‐time PCR (qPCR).

### 2.9 Primers and qPCR

Expression levels of 24 miRNAs were investigated by qPCR. A flow diagram of miRNA investigations is shown in Figure 1, and criteria for selection of miRNAs are listed in Table S1.

For each miRNA assay, primers were designed using a publicly available software “miRprimer”^15^ (Table S2). Quantitative PCR was performed on Mx3005P qPCR system (Sensititre Thermo Scientific Fisher), with qPCR reactions, conditions, melting curve, and PCR efficiency according to Cirera et al.^16^ Quantitative PCR data were pre‐processed using GenEx 6 Pro program (GenEx 6 Pro program, MultiD, Gothenburg, Sweden). Briefly: (1) data were efficiency corrected; (2) data were corrected to the spike‐in miRNA for the stability study (I and II) and normalized to miR23a (stable reference miRNA selected by NormFinder^17^ and GeNorm programs^18^ for the clinical study); (3) both replicates were included in the linear mixed‐effects models (LMMs) in the stability study part I and II, while an average was used for the clinical study; (4) relative quantities (fold changes) were calculated in relation to the sample showing the lowest expression (higher Cq) for the stability study (I and II), or in relation to the average of the control group for the clinical study; and (5) data were log2 transformed before proceeding to statistical analysis.

### 2.10 Statistical analysis

For part I of the stability study the data generated using the Norgen and Qiagen kits were compared by the intraclass correlation coefficient (ICC) to determine similarity among replicates. Based on these results only data obtained using the Qiagen kit was used to analyze the effect of different storage temperatures. Duplicates from 5 samples stored at −20°C, 4 samples stored at −80°C, and 3 samples stored at 4°C were analyzed using LMMs. The LMMs included the log2‐transformed normalized miRNA data as the dependent variable, storage temperature as a fixed effect, while cat and replicate data from the same sample were included as random effects. Principal component analysis, including the miRNAs expressed in all cats in the clinical study, was performed to detect general patterns in miRNA expression profiles. Linear regression was used with the log2‐transformed normalized data for all detected miRNAs in the clinical study as the dependent variables and groups (healthy, PN, or other urological condition) as independent variables. Other variables such as breed (DSH vs Non‐DSH), sex, and age were included as possible confounders and only kept in the final model if *P* < .1 or if the Akaike information criterion was improved by their inclusion. If outliers were detected, a sensitivity analysis was conducted to determine if detected outliers influenced the statistical significance level. Statistical analyses were performed in R version 3.5.0 (April 23, 2018) with a significance level set at 0.05.

## 3 RESULTS

### 3.1 Animals

Twenty‐nine cats were included in the study (Table 1). Twelve cats were characterized as healthy, 5 cats were diagnosed with PN, and 13 cats with a urological condition other than PN. The stability study was performed on urine from the 5 healthy cats initially recruited. As 1 of these cats was later diagnosed with hypertrophic cardiomyopathy, the results from this cat were kept in the analyses of the stability study, but excluded from the analyses of the clinical study.

### 3.2 Stability study

#### 3.2.1 Performance of the RNA isolation kits

A total of 80 cDNA replicates extracted by use of the Norgen Kit (56) and Qiagen kit (24) were used for qPCR. The majority (94%) showed a single peak dissociation curve for each assay confirming 100% specificity and was accepted for further analyses. All *R* ^2^‐values of the standard curve were acceptable (>0.98). PCR efficiencies were acceptable in the range of 80% to 110% for the 3 assays.

Overall, the Qiagen kit showed superior performance compared to the Norgen kit, as demonstrated by lower ICC values and less variation between qPCR replicates (Table S3). Thus, samples extracted with the Qiagen kit were used for the remaining part of the study.

#### 3.2.2 Comparison between storage temperatures

**Figure 2:**
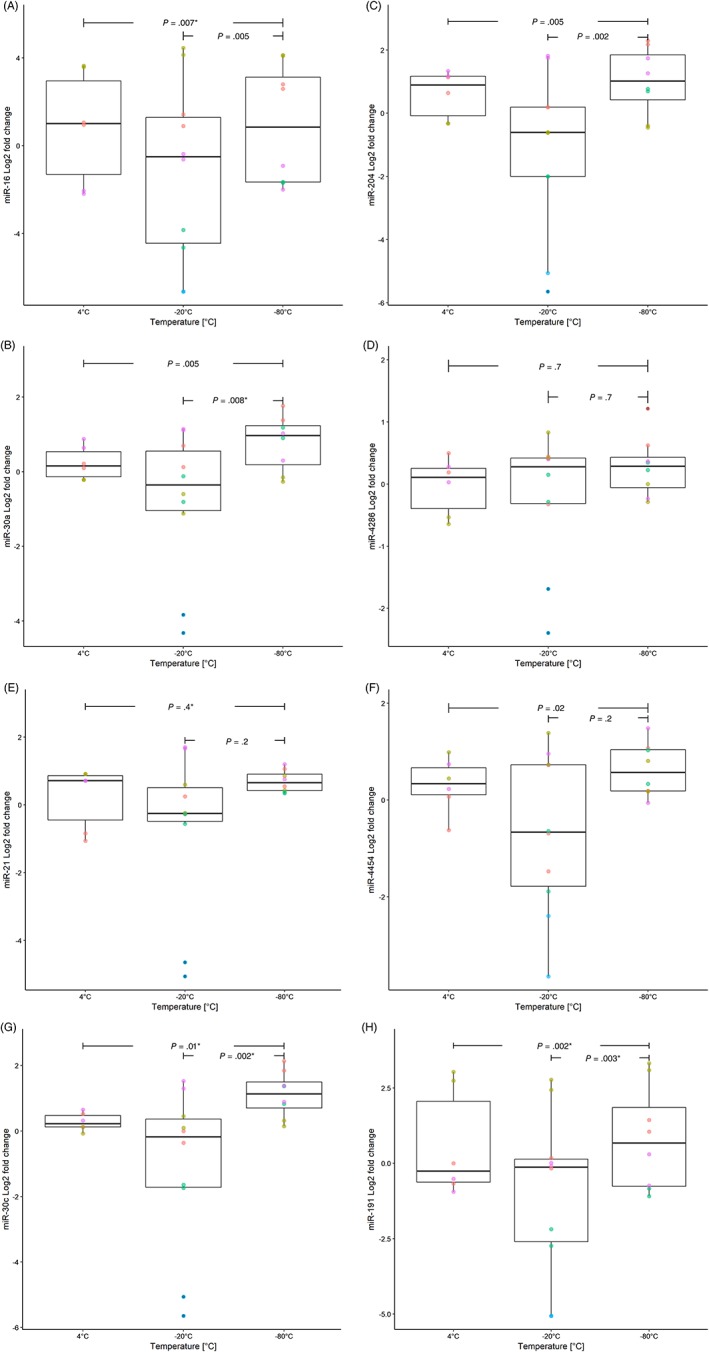
A‐H: The effect of storage temperature (4°C, −20°C, and −80°C) on the yield of the miRNAs used in the clinical study; A, miR‐16; B, miR‐30a; C, miR‐204; D, miR‐4286; E, miR‐21; F, miR‐30c; G, miR‐4454; H, miR‐191—stability study part II. Data represent cDNA duplicates from 5 healthy cats. Colors represent miRNA from the same cat‐sample stored at 3 different temperatures. *Statistically significant

Storage at −80°C resulted in a significantly higher miRNA yield for all 3 miRNAs tested in part I of the stability study (Figure S1) and the majority of miRNAs tested in part II (Figure 2).

#### 3.2.3 Clinical study

**Figure 3:**
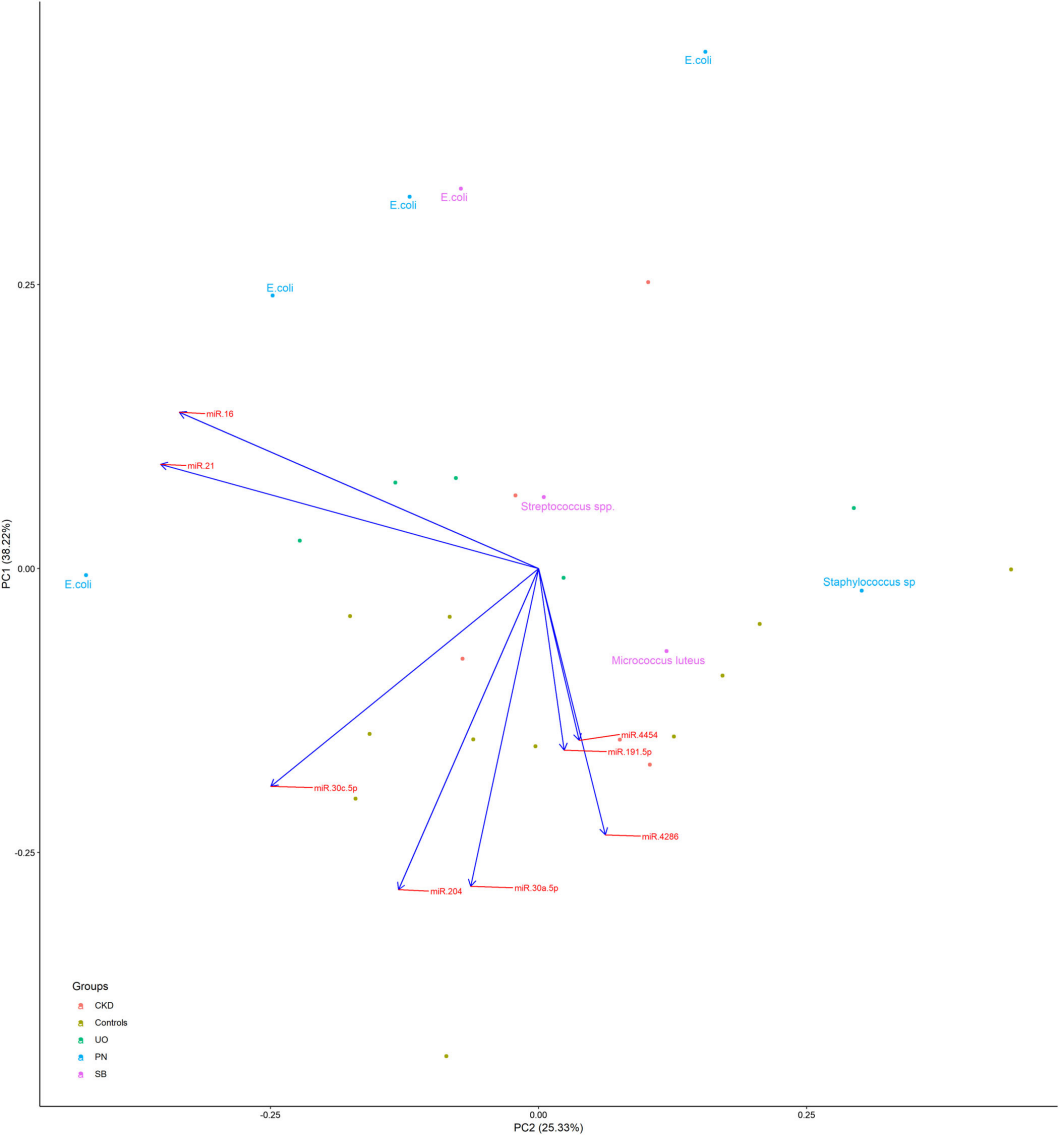
First (PC1) and second (PC2) principal component, from the principal component analysis including the miRNAs (miR‐16, miR‐21, miR‐30c, miR‐204, miR‐30a, miR‐4286, miR‐191, and miR‐4454) from the clinical study, colored by group (chronic kidney disease [CKD]), healthy controls (Controls), ureteral obstruction (UO), pyelonephritis (PN), and subclinical bacteriuria (SB), and the name of the cultured pathogen when relevant

Out of 24 miRNAs investigated, miR‐16, miR‐30a, miR‐204, miR‐4286, miR‐21, miR‐30c, miR‐4454, miR‐191, and miR‐23a (normalizer) were adequately expressed. Plotting the first and second principal components (Figure 3), clustering of data points from the 4 PN cats, and the 1 SB cat with *Escherichia coli* (*E. coli*) was detected. As indicated by the loading plot, this pattern seemed to be due to upregulated miR‐16 and miR‐21 and partially due to downregulation of miR‐204 and miR‐30a.

**Figure 4:**
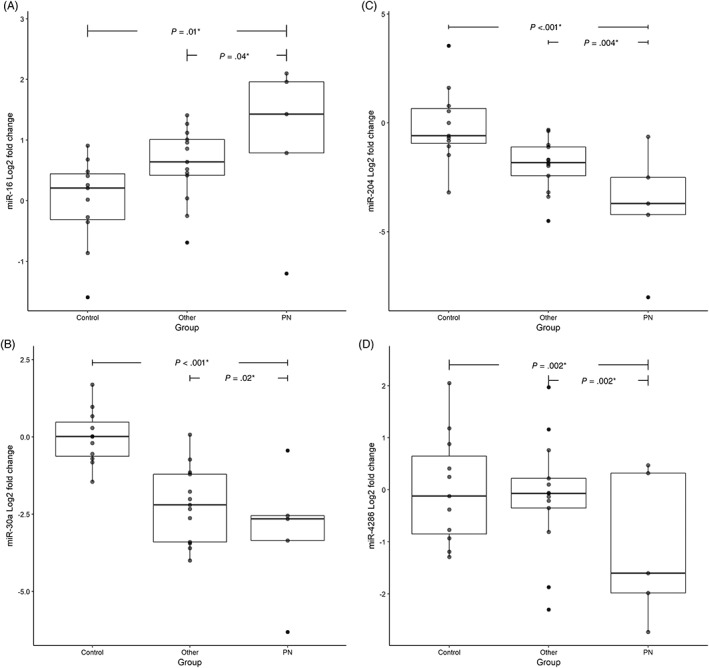
A‐D: Log2 fold changes of the miRNAs A, miR‐16; B, miR‐30a; C, miR‐204; D, miR‐4286 from the clinical study divided by group: pyelonephritis (PN), healthy controls (Controls), and other urological conditions (Other). *Statistically significant

MiR‐16 was significantly upregulated in cats with PN compared with healthy cats (*P* = .01) and cats with other urological conditions (*P* = .04). The sample from the cat with *Staphylococcus*‐associated PN had a cook's distance of >1, indicative of an outlier. MiR‐30a, miR‐4286, and miR‐204 were significantly downregulated in cats with PN compared with healthy cats (*P* < .001, *P* = .02, and *P* < .001, respectively) and compared with cats with other urological conditions (*P* = .02, *P* = .02, and *P* = .004, respectively) (Figure 4). Sex significantly influenced miRNA expression for miR‐30a, miR‐4286, MiR‐30c, and miR‐204, but not miR‐16 (Table S4).

## 4 DISCUSSION

The principal findings of this study are 2‐fold. First, detectability of miRNAs in urine of cats was documented, and, although expression is low, it is relatively stable. Storage temperature influenced urinary miRNA expression levels for the majority of tested miRNAs, with storage temperatures above −80°C leading to a reduced miRNA yield. Second, the clinical study demonstrated clustering of miRNAs from cats with *E. coli* infections (4 PN and 1 SB) and differential expression (upregulation) of miR‐16 in cats with PN compared to healthy cats and cats with other urological conditions. However, it could not be determined if differential expression was PN‐specific, pathogen‐specific (*E. coli*), or both.

The relative stability of urinary miRNAs at different storage conditions confirms the findings of earlier studies^19^ and supports the feasibility of using urinary miRNAs as diagnostic biomarkers. Compared to other RNAs that are rapidly degraded in urine, miRNAs are surprisingly stable, mainly due to their containment in exosomes. However, the effect of storage temperature is not negligible and could potentially skew the results when comparing different groups of animals. In our study, miR‐204, miR‐30a, and miR‐4286 were significantly downregulated in cats with PN compared to healthy cats and cats with other urological conditions. However, downregulation might, at least in part, be due to differences in storage temperatures between samples from different groups of cats, and the importance of these results is therefore uncertain.

Other than storage temperature, miR‐204, miR‐30a, and miR‐4286 expression was also influenced by sex and neuter status, with higher expression levels detected in intact females compared with neutered cats (female and male). However, the influence of sex and neuter status is not expected to have contributed to the downregulation of these miRNAs in cats with PN, as this group had the highest proportion of intact female cats. Future studies must ensure equal storage conditions for all samples and should include a female/female neutered matched control group to minimize the effect of temperature and sex/neuter status on miRNA detection and interpretation.

Urinary miR‐16 was found to be upregulated in cats with PN compared with healthy controls and cats with other urological conditions. As upregulation is opposite the expected effect of the storage temperature in the PN group, the expression of miR‐16 in PN was likely underestimated. It can be speculated whether upregulation of miR‐16 is a pathogen‐directed response rather than, or, in addition to, a PN‐specific response. Indeed the 1 cat with *E. coli*‐associated SB showed very similar results to the PN group, whereas the 1 cat with *Staphylococcus‐*associated PN did not show the same degree of upregulation. Likewise, in the cluster analyses, the cat with *E. coli*‐associated SB, but not the cat with *Staphylococcus*‐associated PN, clustered along with the *E. coli*‐associated PN group. Pathogen‐specific differential expression of miRNAs has been detected in other infections, such as bovine mastitis caused by *E. coli* and *Staphylococcus aureus* 20 and likely involves multiple immunological reactions including toll‐like receptor (TLR) signaling pathways. The underlying pathophysiological explanation to miR‐16 upregulation in our study remains obscure. MiR‐16 is thought to be a critical regulator of TLR‐mediated inflammation and cytokine expression.^21^, ^22^ In people and rodents, host reaction to uropathogenic *E. coli* and UTI phenotype is associated with expression levels of specific chemokine receptors and TLRs,^23^ offering a potential explanation to the link between pyelonephritis, *E. coli*, and miR‐16 expression in our study.

Overlap between individual values of healthy cats, cats with PN and cats with other urological diseases suggests that miR‐16 is not well suited for use as a single discriminatory marker. This is not surprising as miRNAs are often involved in the regulation of multiple disease processes. It will likely require a panel of multiple differentially regulated urinary miRNAs to accurately distinguish PN from other urological conditions.

The limited number of cats in the PN group reflects the strict inclusion criteria and the infrequent performance of pyelocentesis in clinical practice and constitutes the main limitation of this study. Nevertheless, differential expression in cats with PN was detected even with this small number of animals and limited number of miRNAs investigated. Our results suggest potential pathogen‐specific miRNA regulation. This was unexpected, and the study was not designed to account for it. It is of utmost importance that future studies of miRNA expression in cats with PN include a larger number of cats with *Staphylococcus*‐associated PN as well as cats with *E. coli*‐associated infections within (cystitis) and outside the urinary tract in order to discriminate between markers of PN and markers of specific uropathogens. Lastly, the relation between renal function parameters, inflammatory urine sediment and urinary microRNA detection was not investigated in our study. These factors could potentially interact with urinary miRNA detection and should be subject to investigation in future studies including a larger population of cats with PN. In conclusion, this study documented expression of miRNAs in urine of cats, with detection levels influenced by storage temperature. Clustering of cats with *E. coli* and significant upregulation of miR‐16 in cats with PN was documented, suggesting either PN‐specific miRNA regulation, pathogen‐specific miRNA regulation, or both. Together, the results of this study hold promise for the use of urinary miRNAs as future noninvasive biomarkers, and their use in PN in cats deserves further investigation. Directions for future investigations include (1) equal storage temperature of all samples (preferable −80), (2) female/female neutered matched control groups, (3) inclusion of control cats with *E. coli*‐associated infections inside (cystitis) and outside the urinary tract, and (4) pathogen diversity in the PN group.

## CONFLICT OF INTEREST DECLARATION

Authors declare no conflict of interest.

## OFF‐LABEL ANTIMICROBIAL DECLARATION

Authors declare no off‐label use of antimicrobials.

## INSTITUTIONAL ANIMAL CARE AND USE COMMITTEE (IACUC) OR OTHER APPROVAL DECLARATION

Study approval was obtained from the departmental ethical committee at the University of Copenhagen.

## HUMAN ETHICS APPROVAL DECLARATION

Authors declare human ethics approval was not needed for this study.

## Supporting information


**Supplementary Figure 1**(a‐c): The effect of storage temperature (4°C, −20°C, and −80°C) on the yield of the miRNAs miR‐16 (a), miR‐23a (b), and let‐7a (c) ‐stability study part I. Data represent cDNA duplicates from 5 healthy cats. Colors represent miRNA from the same cat‐sample stored at 3 different temperatures. *Statistically significant.

**Supplementary Table 1** Criteria for selection of miRNAs
Supplementary Table 2. Primers of miRNAs tested in the present study
Supplementary Table 3. ICC values
Supplementary Table 4. Illustrating the log2fold mean and SD, and *P*‐value of the urinary miRNAs where sex and neutering was found to be a significant co‐factor in the clinical study
