# Supplementary figures and images for: Stability and profiling of urinary microRNAs in healthy cats and cats with pyelonephritis or other urological conditions
Source: J Vet Intern Med. 2019 Nov 13;34(1):166–75. doi: 10.1111/jvim.15628 (PMC6979273; doi:10.1111/jvim.15628)

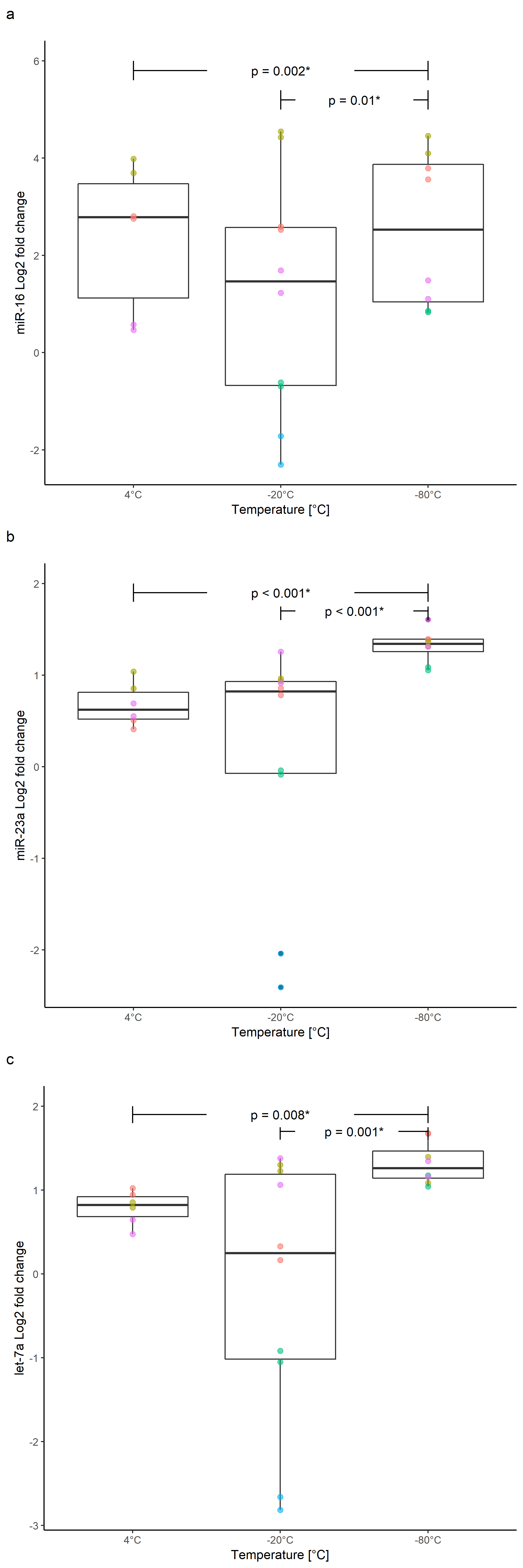

Supplement: Supplementary file 1 — Supplementary Figure 1(a‐c): The effect of storage temperature (4°C, −20°C, and −80°C) on the yield of the miRNAs miR‐16 (a), miR‐23a (b), and let‐7a (c) ‐stability study part I. Data represent cDNA duplicates from 5 healthy cats. Colors represent miRNA from the same cat‐sample stored at 3 different temperatures. *Statistically significant. [file JVIM-34-166-s001.tiff]
