# Supplementary material for: Stability and profiling of urinary microRNAs in healthy cats and cats with pyelonephritis or other urological conditions
Source: J Vet Intern Med. 2019 Nov 13;34(1):166–75. doi: 10.1111/jvim.15628 (PMC6979273; doi:10.1111/jvim.15628)
Supplement: Supplementary file 2 — Supplementary Table 1 Criteria for selection of miRNAs Supplementary Table 2. Primers of miRNAs tested in the present study Supplementary Table 3. ICC values Supplementary Table 4. Illustrating the log2fold mean and SD, and P‐value of the urinary miRNAs where sex and neutering was found to be a significant co‐factor in the clinical study [file JVIM-34-166-s002.pdf]

Supplementary Table 1. Criteria for selection of miRNAs

Criteria for selection	miRNAs
(a) Highly expressed in urine	let-7a, miR-16 and miR-23a
(b) Reported in kidney tissue (cortex and/or medulla) from healthy cats[20]	let-7a, let-7b, miR-16, miR-23a, miR-23b, miR29-c, miR-30a, miR30c, miR99b, miR-126, miR-132, miR155, miR-192, miR-194
(c) Reported in allograft pyelonephritis in people (kidney Tissue)[5]	miR-145, miR-23b, miR-30a, miR-99b, miR-194, miR-200b-3p, miR-204, let-7b, miR-4286, miR-4454
(d) Reported in AKI in humans and/or animals (kidney tissue and/or urine)[7,21]	miR-16, miR-21, miR-30a, miR-146a, miR-155, miR-192
(e) Reported in CKD in humans and/or animals (kidney tissue and/or urine)[7,8,22,23]	let-7a, miR-21, miR23-b, miR-29c, miR-30c, miR-126, miR146a, miR-217
(f) Marker of oncological diseases of the lower urinary tract in dogs[24]	miR-106b, miR-16 (Reported in canine TCC*)

miRNA, microRNA; AKI, acute kidney injury; CKD, chronic kidney disease

Supplementary Table 2. Primers of miRNAs tested in the present study

Name	Mature sequence	Forward primer	Reverse primer
**C.elegans miR-39a-3p**	UCACCGGGUGUAAAUCAGCUUG	GTCACCGGGTGTAAATCAG	CCAGTTTTTTTTTTTTTTTCAAGCTG
**let-7a**	UGAGGUAGUAGGUUGUAUAGUU	GCAGTGAGGTAGTAGGTTGT	GGTCCAGTTTTTTTTTTTTTTTAACTATAC
**let-7b**	UGAGGUAGUAGGUUGUGUGGUU	GTGAGGTAGTAGGTTGTGTG	GGTCCAGTTTTTTTTTTTTTTTAACCA
**miR-16**	UAGCAGCACGUAAAUAUUGGCG	CAGTAGCAGCACGTAAATATTG	CAGTTTTTTTTTTTTTTTCGCCAA
**miR-21**	UAGCUUAUCAGACUGAUGUUGA	GCAGTAGCTTATCAGACTGATG	GGTCCAGTTTTTTTTTTTTTTTCAAC
**miR-23a**	AUCACAUUGCCAGGGAUUU	AGATCACATTGCCAGGGA	GGTCCAGTTTTTTTTTTTTTTTAAATCC
**miR-23b**	UGGGUUCCUGGCAUGCUGAUUU	GGGTTCCTGGCATGCT	GGTCCAGTTTTTTTTTTTTTTTAAATCAG
**miR-29c**	UGACCGAUUUCUCCUGGUGUUC	GTGACCGATTTCTCCTGGT	TCCAGTTTTTTTTTTTTTTTGAACAC
**miR-30a**	UGUAAACAUCCUCGACUGGAAG	GCAGTGTAAACATCCTCGAC	TCCAGTTTTTTTTTTTTTTTCTTCCA
**miR-30c**	UGUAAACAUCCUACACUCUCAGC	CAGTGTAAACATCCTACACTCT	TCCAGTTTTTTTTTTTTTTTGCTG
**miR-99b**	CACCCGUAGAACCGACCUUGCG	CCCGTAGAACCGACCT	TCCAGTTTTTTTTTTTTTTTCGCA
**miR-106b**	UAAAGUGCUGACAGUGCAGAU	GCAGTAAAGTGCTGACAGTG	GGTCCAGTTTTTTTTTTTTTTTATCTG
**miR-126**	CAUUAUUACUUUUGGUACGCG	CGCAGCATTATTACTTTTGGT	GTTTTTTTTTTTTTTTCGCGTACC
**miR-132**	ACCGUGGCUUUCGAUUGUUACU	ACCGTGGCTTTCGATTG	GGTCCAGTTTTTTTTTTTTTTTAGTAAC
**miR-145**	GUCCAGUUUUCCCAGGAAUCCCU	GTCCAGTTTTCCCAGGAATC	AGGTCCAGTTTTTTTTTTTTTTTAGG
**miR-146a**	UGAGAACUGAAUUCCAUGGGUU	GCAGTGAGAACTGAATTCCA	GGTCCAGTTTTTTTTTTTTTTTAACC
**miR-155**	UUAAUGCUAAUCGUGAUAGGGGU	GCAGTTAATGCTAATCGTGATAGG	AGGTCCAGTTTTTTTTTTTTTTTACC
**miR-191**	CAACGGAAUCCCAAAAGCAGCUG	CAACGGAATCCCAAAAGC	AGTTTTTTTTTTTTTTTCAGCTGCT
**miR-192**	CUGACCUAUGAAUUGACAGCC	CAGCTGACCTATGAATTGACA	CCAGTTTTTTTTTTTTTTTGGCTGT
**miR-194**	UGUAACAGCAACUCCAUGUGGA	CAGTGTAACAGCAACTCCA	GTCCAGTTTTTTTTTTTTTTTCCAC
**miR-200b-3p**	UAAUACUGCCUGGUAAUGAUGA	CGCAGTAATACTGCCTGGT	GGTCCAGTTTTTTTTTTTTTTTCATCA
**miR-204**	UUCCCUUUGUCAUCCUAUGCCU	CAGTTCCCTTTGTCATCCTATG	GTCCAGTTTTTTTTTTTTTTTAGGCA
**miR-217**	UACUGCAUCAGGAACUGAUUGGA	AGTACTGCATCAGGAACTGA	GGTCCAGTTTTTTTTTTTTTTTCCA
**miR-4286**	ACCCCACUCCUGGUACC	AGACCCCACTCCTGGT	GGTCCAGTTTTTTTTTTTTTTTGGT
**miR-4454**	GGAUCCGAGUCACGGCACCA	GCAGGGATCCGAGTCAC	GGTCCAGTTTTTTTTTTTTTTTGGT

Legend: Mature sequence used for primer design. Forward and reverse primers used for qPCR.

Supplementary Table 3. ICC values

	ICC		ICC confidence interval 95%	
	Qiagen	Norgen	Qiagen	Norgen
miR-16	0.94	0.59	0.80-0.98	0.15-0.84
miR-23a	0.77	0.58	0.40-0.93	0.29-0.88
let-7a	0.83	0.88	0.52-0.95	0.69-0.95

ICC values for miR-16, miR-23a, and let-7a using the Qiagen and the Norgen kits for miRNA extraction ICC, Intraclass correlation coefficient

Supplementary Table 4. Illustrating the log2fold mean and standard deviation (sd), and p-value of the urinary miRNAs where sex and neutering was found to be a significant co-factor in the clinical study

			n	mean (sd)	p-value
**miR-30a**	Sex and neutering				
		F	7	-0.88 (1.6)	0.01
		FS	11	-1.67 (2.1)	
		F	7	-0.88 (1.6)	0.009
		MN	11	-1.74 (1.0)	
		FS	11	-1.67 (2.1)	0.7
		MN	11	-1.74 (1.0)	
**miR-4286**	Sex and neutering				
		F	7	0.23 (1.2)	0.07
		FS	11	-0.41 (1.4)	
		F	7	0.23 (1.2)	0.04
		MN	11	-0.38 (1.0)	
		FS	11	-0.41 (1.4)	0.6
		MN	11	-0.38 (1.0)	
**miR-30c**	Sex and neutering				
		F	7	0.23 (1.5)	0.04
		FS	11	-0.82 (1.4)	
		F	7	0.23 (1.5)	0.04
		MN	11	-0.89 (1.1)	
		FS	11	-0.82 (1.4)	0.8
		MN	11	-0.89 (1.1)	
**miR-204**	Sex and neutering				

	F	7	-0.92 (2.4)	0.02
	FS	11	-1.84 (2.7)	
	F	7	-0.92 (2.4)	0.02
	MN	11	-1.75 (1.2)	
	FS	11	-1.84 (2.7)	0.7
	MN	11	-1.75 (1.2)	
